# SOMA: A Single Oligonucleotide Mutagenesis and Cloning Approach

**DOI:** 10.1371/journal.pone.0064870

**Published:** 2013-06-04

**Authors:** Thorsten Pfirrmann, Ashwin Lokapally, Claes Andréasson, Per Ljungdahl, Thomas Hollemann

**Affiliations:** 1 Department of Molecular Medicine, Institute for Physiological Chemistry, Martin-Luther University Halle-Wittenberg, Halle, Germany; 2 Department of Molecular Biosciences, The Wenner-Gren Institute, Stockholm University, Stockholm, Sweden; Universidad Miguel Hernández de Elche, Spain

## Abstract

Modern biology research requires simple techniques for efficient and restriction site-independent modification of genetic material. Classical cloning and mutagenesis strategies are limited by their dependency on restriction sites and the use of complementary primer pairs. Here, we describe the Single Oligonucleotide Mutagenesis and Cloning Approach (SOMA) that is independent of restriction sites and only requires a single mutagenic oligonucleotide to modify a plasmid. We demonstrate the broad application spectrum of SOMA with three examples. First, we present a novel plasmid that in a standardized and rapid fashion can be used as a template for SOMA to generate GFP-reporters. We successfully use such a reporter to assess the *in vivo* knock-down quality of morpholinos in *Xenopus laevis* embryos. In a second example, we show how to use a SOMA-based protocol for restriction-site independent cloning to generate chimeric proteins by domain swapping between the two human hRMD5a and hRMD5b isoforms. Last, we show that SOMA simplifies the generation of randomized single-site mutagenized gene libraries. As an example we random-mutagenize a single codon affecting the catalytic activity of the yeast Ssy5 endoprotease and identify a spectrum of tolerated and non-tolerated substitutions. Thus, SOMA represents a highly efficient alternative to classical cloning and mutagenesis strategies.

## Introduction

The study of modern molecular biology requires techniques that facilitate the flexible and targeted manipulation of genetic material. Novel methods for DNA domain shuffling and site-directed or random mutagenesis of genes are critical for advancement in fields of molecular biology like synthetic biology, developmental biology and protein engineering [1]. Site-directed mutagenesis is a commonly used method for the engineering of proteins with wide applications in contemporary biological studies [2], [3]. Advanced methods to manipulate DNA are used to create internal deletions and insertions, to carry out site-directed and random mutagenesis, and to precisely shuffle defined genetic elements. Literature documents a variety of methodological approaches [4]. However, most methods apply PCR with oligonucleotide primer pairs to introduce respective mutations, e.g. Stratagene’s QuikChange™ protocol [5]. This has several drawbacks, e.g. primer pairs carrying desired mutations anneal stronger to each other than to the target sequences. Often this limits the efficiency of the reaction. Moreover, the use of primer pairs may cause the introduction of non-homologous base pairs within the mutagenized codon when generating semi-random mutagenized gene libraries. To circumvent this problem, several alternative methods have been published [6], [7], [8], often with modified primer designs and time consuming additional working steps. An interesting method commercially available as QuikChange Multi™ (Stratagene) involves Pfu-DNA polymerase extension and ligation of plasmids. However, the required composition of the reagents is not published [9] and the method does not employ state-of-the-art reagents such as high-speed proofreading polymerases based on Sso7d fusions [10] commercialized as Phusion High-Fidelity DNA Polymerase (Finnzymes, Thermo Scientific).

Here, we describe the Single-Oligonucleotide Mutagenesis and Cloning Approach (SOMA) that is based on high-speed proofreading Phusion High-Fidelity DNA polymerase extension of a single phosphorylated and mutagenic primer annealed to a plasmid template. The extended DNA is concomitantly ligated by thermostable Taq DNA ligase and the reaction containing single stranded DNA can directly be used to transform *Escherichia coli* after removal of the template plasmid by DpnI digestion. We present three SOMA applications that exemplify the enormous potential of this method.

## Results and Discussion

**Figure 1:**
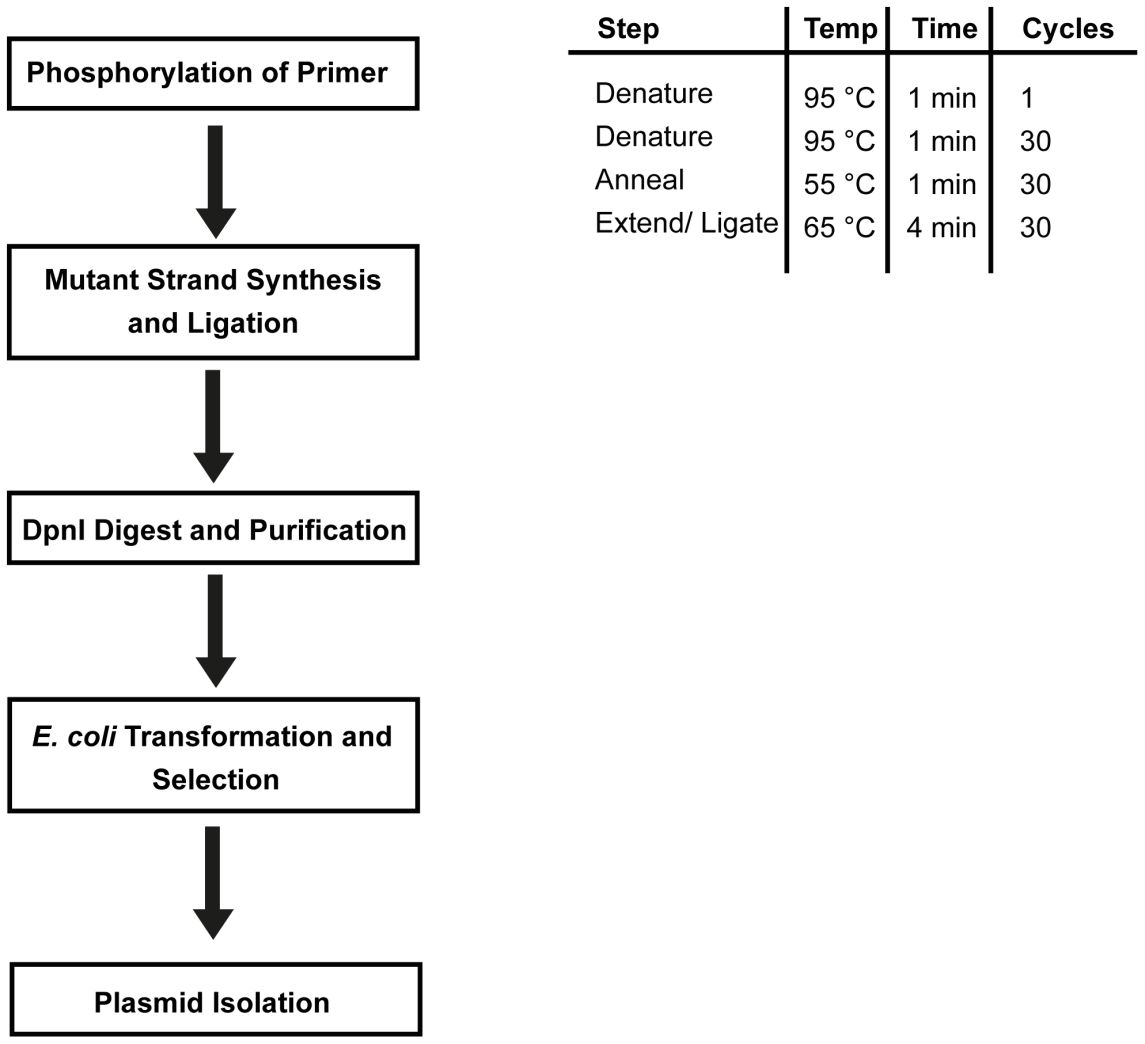
Flow scheme of the SOMA method (left). A mutagenesis primer is phosphorylated 5′ and used for a PCR reaction. Phusion polymerase amplifies the mutant strand, Taq Ligase ligates the nicks during the reaction. A DpnI digest leaves the mutagenized single stranded plasmid that is directly transformed into *E. coli* for selection and plasmid isolation. PCR condition for SOMA (right).

**Figure 2:**
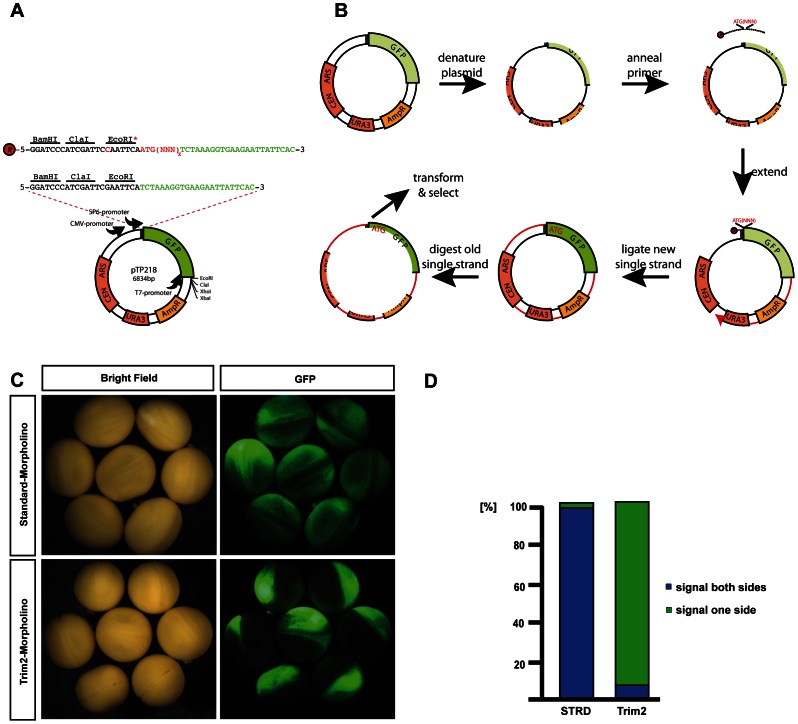
Standard primer for SOMA-construction of a GFP reporter (A). Phosphorylated primer (red P) with diagnostic EcoRI site (red C; EcoRI*), 23 bp of GFP (green sequence) and morpholino target sequence in frame with GFP (NNN)_x_ including ATG (red sequence). Green fluorescent protein (GFP) gene (green); ampicillin resistance gene (Amp^R^; yellow); *URA3* gene, yeast centromere (*CEN*), autonomous replication sequence (*ARS*) (red). Schematic outline of the SOMA method for production of GFP reporters (B). (1) denature pTP218; (2) 5′ phosphorylated single primer anneals; (3) primer is extended to the 5′ end; (4) nicks are ligated; (5) template strand is digested with DpnI; (6) mutated single stranded plasmid transformed into *E. coli* and positive clones identified by lack of EcoRI site. Test of TRIM2 GFP reporter construct *in vivo* (C). Synthetic reporter sense RNA injected into fertilized *Xenopus laevis* embryos at the one-cell stage; TRIM2- or Standard (STRD)-morpholino injected into one blastomere at the two-cell stage. Visualization after 1 day by brightfield or fluorescence microscopy (GFP). 99 of total 101 standard morpholino (STRD) injected embryos showed a GFP signal on both sides (98%); 249 of total 271 Trim2- morpholino injected embryos showed a GFP signal on one side (92%) (right).

SOMA is a technique for the site-directed mutagenesis of plasmids including substitutions, deletions and insertions. Additionally, the insertion feature can be employed to clone and shuffle DNA fragments. We routinely use SOMA to introduce mutations at single and multiple positions with success rates up to 90% depending on the primer design as assessed by diagnostic restriction digestion analysis of individual clones after primary transformation. The basic methodology is outlined in Fig. 1 and a specific application is schematically depicted in Fig. 2B. Briefly, a mutagenic primer complementary to the target sequence is designed to carry the desired mutation. It can either be directly synthesized with a 5′ phosphate group or it can be phosphorylated as described in the Methods section. In a thermocycler the mutagenic primer is annealed to the plasmid template, extended with Phusion High-Fidelity DNA polymerase and the fully extended product is made circular by ligation using Taq DNA ligase. Following 30 cycles of amplification, the template is removed by DpnI digestion and the circular, single stranded mutagenized plasmid is used for *E.coli* transformation. After appropriate selection the plasmids are isolated and subjected to diagnostic restriction digestion or DNA sequencing. Standard thermocycler conditions are presented in Fig. 1. SOMA is based on Phusion High-Fidelity DNA polymerase that is a proofreading polymerase with extremely high extension rates, thus making the method suitable also for very large plasmids. To this end we have successfully mutagenized pBR322-derived plasmids as large as 14.3 kb. Out of 4 clones analyzed, 1 contained the desired substitution mutation as scored by diagnostic restriction analysis facilitated by the introduction of an HaeII restriction site together with the substitution mutation. To demonstrate the versatility of SOMA we present several applications.

### Cloning of *in vivo* GFP Reporter Plasmids using SOMA

Morpholino oligonucleotides (Morpholinos) are anti-sense gene knock-down tools applied in many model organisms most prominently in embryos of *Xenopus* and *Teleostei* for developmental studies [11], [12]. Morpholinos are either applied to block splicing or translation [13], [14]. To inhibit translation, morpholinos are designed to bind around the start codon and block ribosome assembly. The level of impaired translation is best assessed using specific antibodies [11], however, their availability is often restricted and the quality too low to detect endogenous proteins. For this reason we have developed a basic plasmid (pTP218; Table 1) that easily can be modified by an insertion mutagenesis based on SOMA to produce specific GFP-reporters designed to assess morpholino capacity to block translation *in vivo.* The method outline and the standardized primer design are presented in Fig. 2.

**Table 1:** Plasmids used in this study.

Plasmid	Description	Reference
pTP213	pCS2^+^ (URA3) containing CEN/ARS	This work
pTP218	pCS2^+^-GFP (URA3) containing CEN/ARS	This work
pTP224	pEGFP-C1 containing RMND5b (homo sapiens)	This work
pTP225	pEGFP-C1 containing RMND5a (homo sapiens)	This work
pTP233	pEGFP-C1 containing RMND5b_1-420_- RMND5a_421-1173_	This work
pTP234	pEGFP-C1 containing RMND5b_1-729_–RMND5a_730-1173_	This work
pTP157	pRS316 (URA3) containing *HAi-SSY5-GST (I378D)*	This work
pTP158	pRS316 (URA3) containing *HAi-SSY5-GST (I378S)*	This work
pTP159	pRS316 (URA3) containing *HAi-SSY5-GST (I378V)*	This work
pTP160	pRS316 (URA3) containing *HAi-SSY5-GST (I378G)*	This work
pTP165	pRS316 (URA3) containing *HAi-SSY5-GST (I378F)*	This work
pTP167	pRS316 (URA3) containing *HAi-SSY5-GST (I378V)*	This work
pTP170	pRS316 (URA3) containing *HAi-SSY5-GST (I378L)*	This work
pSH120	pRS316 (URA3) containing *HAi-SSY5-GST*	[28]
pAL001	pTP218 containingacagtggtctaggatggccagtgaagcg-GFP	This work

Plasmid pTP218 contains a GFP encoding gene without a start codon downstream of the SP6-promotor (Fig. 2A). A unique EcoRI site is removed during the insertion of the morpholino target sequence in front of GFP by SOMA thereby facilitating the identification of correct clones by diagnostic restrictions (EcoRI). Additionally, the plasmid contains *Saccharomyces cerevisiae* plasmid replication sequences (*CEN/ARS* sequence) and a selection marker (*URA3)* to allow yeast homologous recombination cloning [15].

The standard primer employed to introduce the morpholino target sequences contains homologous regions up- and downstream of the GFP start codon (black/green). An insertion (red) contains a start codon and 25 bp of the morpholino target sequence in frame with GFP.

As an example we have inserted the morpholino target sequence for *Xenopus laevis* ‘Tripartite Motif-containing Protein 2′ (TRIM2) in the GFP open reading frame using SOMA. For functional testing, we employed the SP6 promoter for *in vitro* amplification of synthetic GFP-reporter sense RNA. To assess the knock-down quality of our TRIM2 morpholino, 2.5 ng of RNA was injected into *Xenopus laevis* 1-cell embryos immediately after fertilization to ensure an even distribution of the reporter. At the two-cell stage the embryos were additionally injected with 2.5 pmol of standard morpholino (Figure 2C; upper panel) or TRIM2 morpholino (Figure 2C; lower panel) into one of the two blastomeres. Embryos were harvested at stage 18/19 according to Nieuwkoop and Faber (1967) and photographed (Bright field, GFP). TRIM2-morpholino injected embryos show GFP signal only in one side, standard-morpholino injected ones in both sides of the embryo (Figure 2C). Figure 2D illustrates this phenotype in a bar graph. Of 101 standard morpholino injected eggs, 99 showed a GFP signal in both sides ( = 98%, blue bar). Of 271 TRIM2-morpholino injected eggs, 249 showed no or little signal on the injected side ( = 92%, green bar). Thus, the TRIM2-morpholino efficiently suppresses the translation of the SOMA generated GFP reporter *in vivo*. This demonstrates that pTP218 combined with SOMA can be used to produce customized GFP-reporter constructs for the assessment of morpholino-specificity *in vivo*.

Plasmid pTP218 can also be used to clone N- and C-terminal GFP-fusion products. This can be achieved by conventional cut and paste cloning or by homologous recombination cloning in *Saccharomyces cerevisiae* [15] since the plasmid has yeast replication and selection features. This allows restriction site independent, seamless and fast cloning of overlapping, multiple DNA fragments in a few steps [16]. Since pTP218 (Table 1) is a pCS2^+^ derivative, it contains a functional SP6 promoter, which allows *in vitro* RNA synthesis e.g. for *in vitro* transcription/translation assays and it can be used for transient expression in various cell lines [17].

### Restriction-site Independent Cloning

The ability to rapidly and precisely assemble diverse genetic elements is critical for many fields of modern biology, but perhaps particularly so for the advancement of synthetic biology. There is, despite the dropping prices of synthetic genes, a high demand for methods that allow the restriction-site-independent cloning for the production of recombinant hybrid proteins with novel functions. A vast repertoire of such methods can be found in the literature [18], [19], [20], [21], [22], [23] and each method comes with beneficial and less beneficial properties. A SOMA-based method combined with a regular PCR amplification can be used for restriction-site independent, seamless protein fusions. This allows fast and reliable hybrid gene construction. Briefly, the DNA fragment to be cloned is converted into a PCR product with appropriate plasmid homology at its ends, effectively turning it into a megaprimer that can be introduced into the vector.

**Figure 3:**
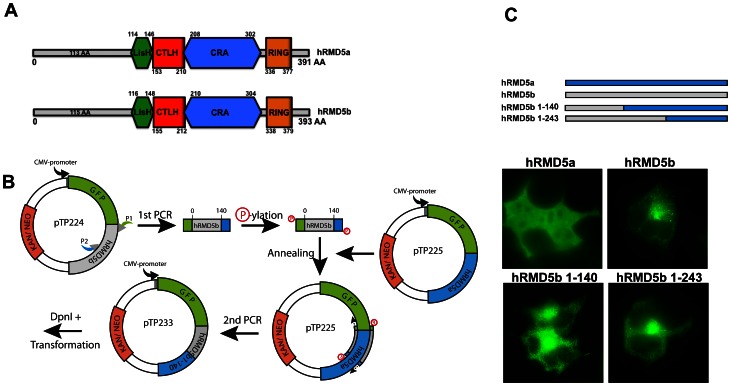
Restriction site independent cloning. (A) Schematic representation of two human RMND5 isoforms (hRMD5a, hRMD5b) with domains; numbers indicate amino acids. (B) Schematic outline of RMND5a/b chimera production. (1) pTP224 as template; (2) PCR with primer P1 and P2 resulting in RMND5b fragment (3) Phosphorylation of 1^st^ PCR product. (4) 5′ phosphorylated PCR product anneals; (5) is extended to the 5′ end; (6) nicks are ligated; (7) template strand is digested with DpnI; (8) product transformed into *E. coli*. Green fluorescent protein (GFP) gene (green); Kanamycine resistance gene (Kan/Neo; orange); RMND5b (grey); RMND5a (blue) (C) Localization of hRMD5a (blue), hRMD5b (grey) and different hybrid fusion (blue/grey) in HEK293 cells. Length of RMD5b fragments indicated as numbers of amino acids (e.g. hRMD5b 1-140).

In Figure 3A we show a schematic representation of the two human ‘Required for Meiotic Nuclear Division 5 Homolog’ isoforms RMND5a (hRMD5a) and RMND5b (hRMD5b) and their protein domains. The similarity of both isoforms is not only reflected in their domain distribution but also on the amino acid residue level with 70% identical residues. We have fused both proteins to the C-terminus of GFP and examined their localization after transfection of HEK293 cells (Fig. 3C). Despite strong identity, both isoforms localize very differently in cells. RMND5a is distributed in the cytosol and in the nucleus of the cell (upper panel, left), whereas RMND5b is mostly present in the cytosol in vesicular structures (upper panel, right). To identify sequence elements responsible for the altered localization, we made several hybrid RMND5 fusion proteins using a SOMA-like method (Fig. 3B). The produced fusion proteins are schematically illustrated in Fig. 3C (upper panel) with RMND5a (hRMD5a; blue bar), RMND5b (hRMD5b; grey bar) and several hybrid proteins thereof. The length of RMND5b fragments in the hybrid proteins is depicted in numbers of amino acids (e.g. hRMD5b 1-140). The SOMA-based method is outlined in Figure 3B. Briefly, several N-terminal RMND5b fragments were PCR-amplified. Primer pairs were chosen to contain 25 bp of homologous region to the RMND5a replacement site (TP231fwd; TP233rev; TP234rev; see Table 2). The resulting PCR products were phosphorylated and used as megaprimers for mutagenesis with a plasmid encoding RMND5a as a template. This specific application exhibited a success rate between 10% and 40%. A similar method dubbed “overlap extension PCR” has been described recently [19]. We find that our modified SOMA-based protocol can be applied for cloning purposes in a similar fashion with high success rates.

**Table 2:** Primers used in this study.

Primer	Description	Reference
TP157fwd	gtattgtgtgaaagattacgataaaaaagctgcaagcgcagtaggcagtattccgtc	This work
TP158fwd	gtattgtgtgaaagattactctaaaaaagctgcaagcgcagtaggcagtattccgtc	This work
TP159fwd	gtattgtgtgaaagattacgttaaaaaagctgcaagcgcagtaggcagtattccgtc	This work
TP160fwd	gtattgtgtgaaagattacggtaaaaaagctgcaagcgcagtaggcagtattccgtc	This work
TP213fwd	atcggtgcgggcctcttcgctattacgccaccgaaaagtgccacctgggt	This work
TP213rev	gtccatatacgccatattgaattggctatgctccttacgcatctgtgcgg	This work
TP218fwd	ttgcaggatcccatcgattcgaattcatctaaaggtgaagaattattcac	This work
TP218rev	tacgactcactatagttctagaggctcgagctatagggagaccggcagatc	This work
AL001fwd	ggatcccatcgattccaattcacagtggtctaggatggccagtgaagcgtctaaaggtgaagaattattcac	This work
TP224fwd	catcatctcgagaagagcagtgtgcgtgcgtgg	This work
TP224rev	catcataagcttgagcagaatatgatgcgtttcccatctg	This work
TP225fwd	catcatctcgagaagatcagtgcgtgacggtg	This work
TP225fwd	catcataagcttgagtcagaaaaatatctgtttggcatctc	This work
TP231fwd	gctgtacaagtccggactcagatcacgagaagagcagtgtgcgtgcgtgg	This work
TP233rev	cttgggtctacagaaagaccagattcctgacacagctcctcggccacgc	This work
TP234rev	gaacatatggtgagttctcaatcccttgtctcaggtacaccaggctgccc	This work
D378Xfwd	gtattgtgtgaaagattacnnnaaaaaagctgcaagcgcagtaggcagtattccgtc	This work

### Generation of a Gene Library with Randomized Site-directed Mutations using SOMA

Understanding protease specificity is a challenging task. Standard nomenclature defines the first amino acids of the C-terminal cleavage fragment as P1’–P4’, the last ones of the N-terminal fragment as P1–P4 [24]. Often proteases accept several amino acids at these positions and their determination requires laborious screening methods [25]. SOMA can be used to generate semi-random mutagenized gene libraries, which have the potential to greatly facilitate such screens. Experimentally derived information regarding protease cleavage sites can be applied to predict novel protease substrates by *in silico* approaches [26].

**Figure 4:**
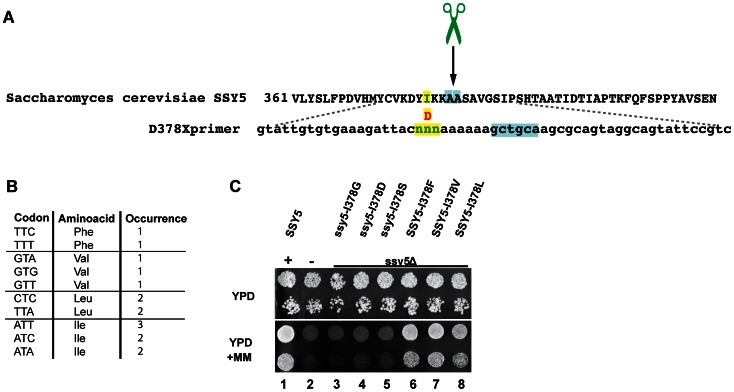
Generation of a semi-random mutagenized gene library using SOMA. (A) Ssy5 sequence with autolytic processing site (scissors) between A381 and A382 (blue). Replacement of conserved isoleucine378 (I; green) with aspartate (D; red) inactivates Ssy5. Primer D378X with random codon (NNN) theoretically capable of encoding all amino acids at position 378 (green). (B) Table with active D378 substitutions recovered in plasmids selected based on their ability to confer Ssy5-dependent growth on YPD+MM. (C) Dilutions of strain HKY77 (ssy5Δ) carrying pSH120 (SSY5; +), pRS316 (empty plasmid; -), I378G, I378D, I378S, I378F, I378V, or I378L.

The activity of the *S. cerevisiae* Ssy5 protease is regulated in response to the availability of extracellular amino acids [27]. During its maturation, Ssy5 cleaves itself between alanine381 and alanine382 (Figure 4A, scissors). This is a requisite event for subsequent amino acid induced activation of the endoproteolytic activity of Ssy5 by proteolytic removal of the inhibitory N-terminal domain [28]. The transcription factors Stp1 and Stp2 are the only other known substrates of Ssy5, however, the cleavage site in these substrates is not known. We noted a highly conserved isoleucine (I; yellow) at position 378 of Ssy5 (Figure 4A). Based on the proximity of a conserved residue to the autolytic processing site (P4’ position), we applied SOMA to produce a gene library randomly mutagenized specifically at codon I378.

Briefly, a SSY5 specific primer with three consecutive random basepairs (NNN) at the codon I378 was synthesized and employed for SOMA using a template plasmid encoding inactive *ssy5*-I378D (Figure 4A; D378Xprimer). After *E. coli* transformation, ∼1000 colonies were collected from the selection media and incubated for an hour at 37°C in LB medium before plasmids were isolated. These were subsequently used as a gene library and directly transformed into a *ssy5Δ* deletion strain. Plasmids carrying mutant alleles of SSY5 with restored activity were selected by their capacity to grow on YPD+MM [28]. Sixteen plasmids were isolated and sequenced as described in the Methods section. A list of the recovered and active mutations, including their codons, amino acid substitutions and occurrence, is shown in Figure 4 (lower panel); seven D378I (wild type), four D378L, three D378V and two D378F substitutions were recovered.

We conclude that SOMA works well to generate a gene library with a randomized codon. In principle it is possible to extend the DNA sequence targeted for mutagenesis but at the cost of coverage. Similarly, multiple mutagenic primers can be employed simultaneously to randomly mutagenize several positions in the same plasmid. When combined with error-prone PCR to generate megaprimers SOMA can be used to randomly mutagenize a larger region. Such approaches will be beneficial for e.g. the development of novel enzymes with for example altered activities, stability or specificity.

Taken together, with these experiments we demonstrate the broad practical utility of SOMA. The SOMA method differs from the commonly used QuikChange™ (Stratagene) method in the use of a single oligonucleotide and ligation of the extended product with a thermostable DNA ligase. SOMA is different to the commercially available QuikChange Multi™ (Stratagene) protocol since SOMA relies on recent advances such as state-of-the-art high speed proofreading DNA polymerase and employs a thermostable DNA ligase.

## Materials and Methods

### Oligonucleotides and Plasmids

Plasmids and primers used in this work are listed in Table 1 and Table 2, respectively and are available upon request. pTP213 was made by yeast homologous recombination with SalI linearized pCS2^+^ and a PCR product obtained with the primers TP213fwd, TP213rev and pRS316 as a template. pTP218 was obtained by yeast homologous recombination with EcoRI/XhoI linearized pTP213 and a GFP encoding PCR fragment generated with TP218fwd, TP218rev from pYMN25. Plasmid pTP224 contains human RMND5b amplified from cDNA (clone BC009911; Open Biosystems) with TP224fwd and TP224rev, inserted via XhoI and HindIII into pEGFP-C1 (Clontech). Accordingly, pTP225 contains RMND5a amplified from cDNA (clone BC047668; Open Biosystems). Both plasmids pTP233 and pTP234 are pTP225 derivatives containing chimeric gene fusions of RMND5a and RMND5b, generated with TP231fwd and TP233rev or TP234, respectively (Figure 3 B). pAL001 is a pTP218 derivative that contains a SOMA generated insertion (Figure 2). The plasmids pTP157, pTP158, pTP159, pTP160, pTP165, pTP167 and pTP170 contain different SOMA generated point mutations at position I378 (Figure 4).

### Organisms and Maintenance

Xenopus: Frogs were obtained from commercial suppliers (NASCO, USA). Production and rearing of embryos was performed as described previously [29]. Embryos were maintained at 15°C and staged according to Nieuwkoop and Faber [30]. All procedures were performed according to guidelines set by the German animal use and care laws (Tierschutzgesetz) and approved by the German state administration Saxony-Anhalt (Projekt/AZ: 42502-3-600 MLU).

Yeast: Yeast extract-peptone-dextrose (YPD) medium was prepared as described previously [31]. Sensitivity to 2-{[({[(4-methoxy-6-methyl)-1,3,5-triazin-2-yl]-amino}carbonyl)amino-]-sulfonyl}-benzoic acid (MM; 450 µg/ml) on complex medium is described elsewhere [32]. Strain HKY77 (MATa lys2201 ura3-52 ssy52::hisG) is an isogenic descendant of AA255/PLY115 [33].

Cell Line: HEK293 cells were maintained in Dulbecco’s modified Eagle’s medium supplemented with 10% fetal calf serum (v/v). Transfections were performed using Lipofectamin 2000 (Invitrogen).

### Capped mRNA and Morpholino Injections

Capped Mo-GFP m-RNA was generated using the mMESSAGE mMACHINE kit (Ambion, Austin, TX). KpnI linearized pAL001 was used as a template for SP6 transcription and 5 nl of capped mRNA (∼2.5 ng) were injected into the 1-cell stage embryo. 25-mer morpholinos (MOs; Gene Tools, LLC Philomath, Oregon) were designed to target the ATG translation start site for Trim2 (NM_001092023) mRNA transcripts. A mismatch standard MO was used as control. Both were injected (2.5 pmol) into one blastomere of 2-cell stage Mo-GFP mRNA injected embryos.

### Oligonucleotide Phosphorylation

Oligonucleotides were phosphorylated prior to SOMA using T4 polynucleotide kinase (NEB) according to the manufacturer. Briefly, 4 mM oligonucleotide primer was phosphorylated for 30 min at 37°C with the corresponding buffers and a final concentration of 1 mM ATP. The reaction was stopped by heat inactivation for 15 min at 65°C. Phosphorylated primers or PCR products were directly used for mutagenesis reactions.

### Single Oligonucleotide Mutagenesis and Cloning Approach (SOMA)

Primers contain the mutation flanked by 20–25 bp of homologous region. Alternatively, for restriction site independent cloning, primer pairs are flanked by 20–25 bp of homologous region to the target DNA. A typical 50 µl reaction contains 0.2 mM primer, 100 ng template DNA, 1 mM NAD^+^ (Sigma-Aldrich), 1 µl High-Fidelity DNA Polymerase (Finnzymes, Thermo Scientific), 1 µl Taq-Ligase (NEB), 10 µl HF-buffer (NEB) and 0,2 mM dNTPs. After an initial denaturing step (1 min at 95°C), DNA was amplified 30 cycles (95°C–1min; 55°C –1 min; 65°C –4 min). The extension time was standardly set to 4 minutes to facilitate the completion of both the polymerase extension and the ligation reaction. However, extension times for particular plasmids may have to be optimized. Afterwards 5 µl of DpnI digestion buffer was directly added (Fast Digest; Fermentas) and template DNA was DpnI digested. After purification (Qiagen PCR purification kit, Qiagen, Hilden) DNA was transformed into *E. coli*.

### Plasmid Isolation from Saccharomyces Cerevisiae

A yeast colony was resuspended in 0.2 ml P1 buffer (QIAprep, Qiagen) and pretreated with Zymolyase (ZymoResearch) for 1 h at 37°C. Further steps were performed as described by the manufacturer (QIAprep, Qiagen). First 0.3 ml P2-buffer was added for cell lysis, then 0.42 ml N3-buffer was added and cell debris removed by centrifugation (10 min, 13000 rpm). The supernatant was applied to spin columns and plasmid DNA was eluted with 30 µl H_2_O after washing (PB- and PE-buffer).

## References

[1] Peccoud J, Isalan M (2012) The PLOS ONE synthetic biology collection: six years and counting. PLoS One 7: e43231. 22916228 10.1371/journal.pone.0043231 PMC3419720

[2] Pleiss J (2011) Protein design in metabolic engineering and synthetic biology. Curr Opin Biotechnol 22: 611–617. 21514140 10.1016/j.copbio.2011.03.004

[3] Antikainen NM, Martin SF (2005) Altering protein specificity: techniques and applications. Bioorg Med Chem 13: 2701–2716. 15781382 10.1016/j.bmc.2005.01.059

[4] Zawaira A, Pooran A, Barichievy S, Chopera D (2012) A discussion of molecular biology methods for protein engineering. Mol Biotechnol 51: 67–102. 21959889 10.1007/s12033-011-9448-9

[5] Braman J, Papworth C, Greener A (1996) Site-directed mutagenesis using double-stranded plasmid DNA templates. Methods Mol Biol 57: 31–44. 8849992 10.1385/0-89603-332-5:31

[6] Edelheit O, Hanukoglu A, Hanukoglu I (2009) Simple and efficient site-directed mutagenesis using two single-primer reactions in parallel to generate mutants for protein structure-function studies. BMC Biotechnol 9: 61. 19566935 10.1186/1472-6750-9-61 PMC2711942

[7] Kirsch RD, Joly E (1998) An improved PCR-mutagenesis strategy for two-site mutagenesis or sequence swapping between related genes. Nucleic Acids Res 26: 1848–1850. 9512562 10.1093/nar/26.7.1848 PMC147442

[8] Zheng L, Baumann U, Reymond JL (2004) An efficient one-step site-directed and site-saturation mutagenesis protocol. Nucleic Acids Res 32: e115. 15304544 10.1093/nar/gnh110 PMC514394

[9] Hogrefe HH, Cline J, Youngblood GL, Allen RM (2002) Creating randomized amino acid libraries with the QuikChange Multi Site-Directed Mutagenesis Kit. Biotechniques 33: 1158–1160, 1162, 1164–1155. 10.2144/02335pf01 12449398

[10] Wang Y, Prosen DE, Mei L, Sullivan JC, Finney M, et al (2004) A novel strategy to engineer DNA polymerases for enhanced processivity and improved performance in vitro. Nucleic Acids Res 32: 1197–1207. 14973201 10.1093/nar/gkh271 PMC373405

[11] Bill BR, Petzold AM, Clark KJ, Schimmenti LA, Ekker SC (2009) A primer for morpholino use in zebrafish. Zebrafish 6: 69–77. 19374550 10.1089/zeb.2008.0555 PMC2776066

[12] Eisen JS, Smith JC (2008) Controlling morpholino experiments: don’t stop making antisense. Development 135: 1735–1743. 18403413 10.1242/dev.001115

[13] Summerton J, Weller D (1997) Morpholino antisense oligomers: design, preparation, and properties. Antisense Nucleic Acid Drug Dev 7: 187–195. 9212909 10.1089/oli.1.1997.7.187

[14] Morcos PA (2007) Achieving targeted and quantifiable alteration of mRNA splicing with Morpholino oligos. Biochem Biophys Res Commun 358: 521–527. 17493584 10.1016/j.bbrc.2007.04.172

[15] Ma H, Kunes S, Schatz PJ, Botstein D (1987) Plasmid construction by homologous recombination in yeast. Gene 58: 201–216. 2828185 10.1016/0378-1119(87)90376-3

[16] Benders GA, Noskov VN, Denisova EA, Lartigue C, Gibson DG, et al (2010) Cloning whole bacterial genomes in yeast. Nucleic Acids Res 38: 2558–2569. 20211840 10.1093/nar/gkq119 PMC2860123

[17] Turner DL, Weintraub H (1994) Expression of achaete-scute homolog 3 in Xenopus embryos converts ectodermal cells to a neural fate. Genes Dev 8: 1434–1447. 7926743 10.1101/gad.8.12.1434

[18] Benoit RM, Wilhelm RN, Scherer-Becker D, Ostermeier C (2006) An improved method for fast, robust, and seamless integration of DNA fragments into multiple plasmids. Protein Expr Purif 45: 66–71. 16289702 10.1016/j.pep.2005.09.022

[19] Bryksin AV, Matsumura I (2010) Overlap extension PCR cloning: a simple and reliable way to create recombinant plasmids. Biotechniques 48: 463–465. 20569222 10.2144/000113418 PMC3121328

[20] Shuldiner AR, Tanner K, Scott LA, Moore CA, Roth J (1991) Ligase-free subcloning: a versatile method to subclone polymerase chain reaction (PCR) products in a single day. Anal Biochem 194: 9–15. 1651068 10.1016/0003-2697(91)90144-i

[21] Zuo P, Rabie BM (2010) One-step DNA fragment assembly and circularization for gene cloning. Curr Issues Mol Biol 12: 11–16. 19494420

[22] Geu-Flores F, Nour-Eldin HH, Nielsen MT, Halkier BA (2007) USER fusion: a rapid and efficient method for simultaneous fusion and cloning of multiple PCR products. Nucleic Acids Res 35: e55. 17389646 10.1093/nar/gkm106 PMC1874642

[23] Zhu B, Cai G, Hall EO, Freeman GJ (2007) In-fusion assembly: seamless engineering of multidomain fusion proteins, modular vectors, and mutations. Biotechniques 43: 354–359. 17907578 10.2144/000112536

[24] Schechter I, Berger A (1967) On the size of the active site in proteases. I. Papain. Biochem Biophys Res Commun 27: 157–162. 6035483 10.1016/s0006-291x(67)80055-x

[25] Diamond SL (2007) Methods for mapping protease specificity. Curr Opin Chem Biol 11: 46–51. 17157549 10.1016/j.cbpa.2006.11.021

[26] Song J, Tan H, Perry AJ, Akutsu T, Webb GI, et al (2012) PROSPER: An Integrated Feature-Based Tool for Predicting Protease Substrate Cleavage Sites. PLoS One 7: e50300. 23209700 10.1371/journal.pone.0050300 PMC3510211

[27] Pfirrmann T, Ljungdahl PO (2012) Chapter 685– Ssy5 Peptidase: A Chymotrypsin-Like Signaling Protease in Yeast. In: Handbook of proteolytic enzymes. Academic Press: Elsevier.

[28] Pfirrmann T, Heessen S, Omnus DJ, Andréasson C, Ljungdahl PO (2010) The prodomain of Ssy5 protease controls receptor-activated proteolysis of transcription factor Stp1. Mol Cell Biol 30: 3299–3309. 20421414 10.1128/MCB.00323-10 PMC2897576

[29] Gurdon JB (1977) Methods for nuclear transplantation in amphibia. Methods Cell Biol 16: 125–139. 329056 10.1016/s0091-679x(08)60096-5

[30] Faber J, Nieuwkoop PD (1967) Normal Table of Xenopus Laevis (Daudin); a Systematical and Chronological Survey of the Development from Fertilized Egg Till the End of Metamorphosis.: Amsterdam: North Holland.

[31] Andréasson C, Ljungdahl PO (2002) Receptor-mediated endoproteolytic activation of two transcription factors in yeast. Genes Dev 16: 3158–3172. 12502738 10.1101/gad.239202 PMC187503

[32] Poulsen P, Lo Leggio L, Kielland-Brandt MC (2006) Mapping of an internal protease cleavage site in the Ssy5p component of the amino acid sensor of Saccharomyces cerevisiae and functional characterization of the resulting pro- and protease domains by gain-of-function genetics. Eukaryot Cell 5: 601–608. 16524914 10.1128/EC.5.3.601-608.2006 PMC1398070

[33] Forsberg H, Ljungdahl PO (2001) Genetic and biochemical analysis of the yeast plasma membrane Ssy1p-Ptr3p-Ssy5p sensor of extracellular amino acids. Mol Cell Biol 21: 814–826. 11154269 10.1128/MCB.21.3.814-826.2001 PMC86673

